# Norepinephrine-Induced Adrenergic Activation Strikingly Increased the Atrial Fibrillation Duration through β1- and α1-Adrenergic Receptor-Mediated Signaling in Mice

**DOI:** 10.1371/journal.pone.0133664

**Published:** 2015-07-23

**Authors:** Kenji Suita, Takayuki Fujita, Nozomi Hasegawa, Wenqian Cai, Huiling Jin, Yuko Hidaka, Rajesh Prajapati, Masanari Umemura, Utako Yokoyama, Motohiko Sato, Satoshi Okumura, Yoshihiro Ishikawa

**Affiliations:** 1 Cardiovascular Research Institute, Yokohama City University Graduate School of Medicine, Yokohama, Japan; 2 Department of Physiology, Aichi Medical University School of Medicine, Aichi, Japan; 3 Department of Physiology, Tsurumi University School of Dental Medicine, Yokohama, Japan; University at Buffalo, UNITED STATES

## Abstract

**Background:**

Atrial fibrillation (AF) is the most common arrhythmias among old people. It causes serious long-term health problems affecting the quality of life. It has been suggested that the autonomic nervous system is involved in the onset and maintenance of AF in human. However, investigation of its pathogenesis and potential treatment has been hampered by the lack of suitable AF models in experimental animals.

**Objectives:**

Our aim was to establish a long-lasting AF model in mice. We also investigated the role of adrenergic receptor (AR) subtypes, which may be involved in the onset and duration of AF.

**Methods and Results:**

Trans-esophageal atrial burst pacing in mice could induce AF, as previously shown, but with only a short duration (29.0±8.1 sec). We found that adrenergic activation by intraperitoneal norepinephrine (NE) injection strikingly increased the AF duration. It increased the duration to more than 10 minutes, i.e., by more than 20-fold (656.2±104.8 sec; P<0.001). In this model, a prior injection of a specific β1-AR blocker metoprolol and an α1-AR blocker prazosin both significantly attenuated NE-induced elongation of AF. To further explore the mechanisms underlying these receptors’ effects on AF, we assessed the SR Ca^2+^ leak, a major trigger of AF, and consequent spontaneous SR Ca^2+^ release (SCR) in atrial myocytes. Consistent with the results of our in-vivo experiments, both metoprolol and prazosin significantly inhibited the NE-induced SR Ca^2+^ leak and SCR. These findings suggest that both β1-AR　and α1-AR may play important roles in the development of AF.

**Conclusions:**

We have established a long-lasting AF model in mice induced by adrenergic activation, which will be valuable in future AF study using experimental animals, such as transgenic mice. We also revealed the important role of β1- and α1-AR-mediated signaling in the development of AF through in-vivo and in-vitro experiments.

## Introduction

Atrial fibrillation (AF) is the most common arrhythmias, especially among elderly people, and causes harmful effects to the patients [1,2]. The lifetime risk of AF for middle-aged people has been estimated to be greater than 20% [3,4], and the prevalence of AF is reported to be increasing in developed countries [5]. In addition to uncomfortable chest symptoms and reduction of cardiac function, AF patients may face a strikingly increased risk of stroke, due to thrombus formation in left atrium. It is thus recommended that such patients should take anti-coagulant medication. However, the adverse complication of the anticoagulant therapy, such as bleeding, is a major clinical problem. All of these issues significantly affect patients’ quality of life. There have been many animal and human studies aimed at reducing the risk of AF to minimize its harmful consequences as suggested in clinical guide lines [6]. However, animal studies of AF, in particular, have been hampered by the lack of suitable AF model because the known AF model has only a short AF duration of seconds [7], not of minutes. Thus it is difficult to evaluate the effect of drug in detail with such a short duration.

Numerous studies have demonstrated that activity of the autonomic nervous system is closely involved in the onset and maintenance of AF [8]. However, the molecular mechanism of autonomic activation-induced AF has not yet been fully elucidated, as the autonomic nervous system regulates the function of cardiomyocytes in a highly complex manner. In addition, research has been hindered by the absence of useful animal models for autonomic activation-induced AF. In this study, we have established a long-lasting AF in mice by the use of trans-esophageal pacing. We will demonstrate that intraperitoneal NE injection strikingly and reliably elongated the duration of atrial burst pacing-induced AF in mice. We have also determined the role of AR subtypes involved in adrenergic activation-induced AF in our model. Because NE activates both α- and β-adrenergic receptors, either or both may play an important role. To further explore the mechanisms by which abovementioned receptors-mediated signaling affect the duration of AF, we assessed the sarcoplasmic reticulum (SR) Ca^2+^ leak, which is known to be a major trigger for AF, and the consequent spontaneous SR Ca^2+^ release (SCR) in mouse atrial myocytes. Our results have indicated that not only β1-AR but also α1-AR-mediated signaling are involved in the NE-induced SR Ca^2+^ leak and SCR as well as maintenance of AF.

## Materials and Methods

### Animals

Male C57BL/6 mice aged 11–12 weeks were purchased from Japan SLC (Shizuoka, Japan). Standard food and water were provided ad libitum to mice. Twelve- to 14-week-old male mice were used for the experiments performed in this study. All animal experiments were approved by the Animal Care and Use Committee of Yokohama City University School of Medicine.

### Induction of atrial fibrillation

Simple and minimally invasive AF models have been established in small animals such as rats and mice. We induced AF using rapid transesophageal atrial pacing according to previously reported methods with some modifications [7]. Briefly, mice were anesthetized by means of isoflurane inhalation (1.5–2.0% for maintenance). A 1.1 French octapolar catheter with eight 0.5-mm circular electrodes and an interelectrode distance of 1 mm (EPR800; Millar Instruments, Houston, TX, USA) was carefully advanced through the esophagus of each mouse. The catheter was placed at the site with the lowest threshold for atrial capture [9]. To ensure the correct position of the pacing catheter, atrial capture with 1:1 atrioventricular conduction was documented prior to the burst pacing period [10]. Transesophageal atrial burst pacing was then conducted for 10 seconds at a stimulation amplitude of 1.5 mA with 10 msec cycle lengths and a pulse width of 3 mA.

### Drug treatment

All the reagents used in this study were purchased from Sigma Aldrich (St. Louis, MO, USA) unless described otherwise. For sympathetic activation, norepinephrine (NE) bitartrate dissolved in natural saline (Otsuka Pharmaceutical, Tokyo, Japan) was intraperitoneally injected 10 minutes before the induction of AF. For the selective blockade of adrenergic receptor (AR), mice were intraperitoneally injected with either 2 mg/kg of metoprolol (a β1-AR-selective antagonist), 1 mg/kg of prazosin (an α1-AR selective antagonist), or natural saline 45 minutes before NE administration.

### Isolation of atrial myocytes

Atrial myocytes were prepared from adult mice as previously described with some modifications [11,12]. Briefly, C57BL/6 mice at 12–14 weeks of age were anesthetized by intraperitoneal injection of pentobarbital (2.3 mg per mouse) with heparin (150 units per mouse). The heart was excised, and the aorta was cannulated and perfused with 2 mL of modified Joklik’s minimal essential medium (JMEM) (Life Technologies, Carlsbad, CA, USA) consisting of 113 mM NaCl, 4.7 mM KCl, 0.6 mM KH_2_PO_4_, 0.6 mM Na_2_HPO_4_, 1.2 mM MgSO_4_, 12 mM NaHCO_3_, 20 mM D-glucose, 10 mM HEPES, 30 mM taurine, 2 mM creatinine, 2 mM carnitine and 5 mM butanedione monoxime (pH 7.4). The atria were cut into several small pieces and incubated in JMEM containing 0.02 mg/mL Liberase TH (Roche, Indianapolis, IN, USA) with occasional agitation for 60 minutes followed by gentle trituration for 5 minutes at 37°C. The same volume of JMEM with 1% (w/v) BSA was added and the suspension was filtered through a 100 μm mesh (BD, Franklin Lakes, NJ, USA). Cells were precipitated by centrifugation for 2 minutes at 40 x *g* and the pellet was gently resuspended in JMEM with 1% (w/v) BSA. Ca^2+^ reproduction was gradually performed to a concentration of 1.25 mM. Myocytes were precipitated again and resuspended in attaching media consisting of Medium 199 (Life Technologies) with 4% (v/v) FBS and 1% (v/v) penicillin/streptomycin (Wako). The cells were then plated onto laminin-coated glass cover-slips and incubated at 37°C in humidified air with 5% CO_2_ for 1 hour. Finally, attaching media was exchanged for maintaining media (Medium 199 containing 1% BSA and 1% penicillin/streptomycin). The prepared myocytes were bathed in maintaining media at 37°C before Ca^2+^ transient was measured.

### Measurement of Ca^2+^ transient

The measurement of Ca^2+^ transient was performed according to previously reported protocols with several modifications [11,13]. All experiments were performed at room temperature. Myocytes were loaded with 5 μM fluo-4 AM (Dojindo, Kumamoto, Japan) in normal Tyrode solution (140 mM NaCl, 5 mM KCl, 1 mM MgCl_2_, 10 mM glucose and 10 mM HEPES, pH 7.4 adjusted with NaOH) containing 1.8 mM Ca^2+^ for 15 minutes. Cells were washed twice with normal Tyrode solution and transferred to a chamber equipped with platinum electrodes. The chamber was placed on a Ti2000 confocal microscope system (Nikon, Tokyo, Japan) in a dark room. To detect SCRs, the external solution was rapidly switched from normal Tyrode to 0Na^+^/0Ca^2+^ Tyrode (140 mM LiCl, 5 mM KCl, 1 mM MgCl_2_, 10 mM glucose, 1 mM EGTA, and 10 mM HEPES adjusted pH to 7.4 with LiOH). Myocytes were bathed for 30 seconds in 0Na^+^/0Ca^2+^ Tyrode solution, and SCRs were counted during this period. After SCR measurement, diastolic Ca^2+^ leak from the SR was estimated by a quick treatment with tetracaine. The ryanodine receptor (RYR) was rapidly and reversibly blocked by 1 mM tetracaine, causing Ca^2+^ uptake from the cytosol into the SR. The tetracaine-dependent shift of Ca^2+^ from the cytosol to the SR was considered to be proportional to the SR Ca^2+^ leak [14]. Finally, 10 mM caffeine was rapidly applied for the estimation of SR Ca^2+^ content.

For drug-treated samples, myocytes were pre-incubated with 1 μM of AR antagonist (prazosin or metoprolol) and 1 μM of NE for 5 minutes prior to the measurement of Ca^2+^ transient. The protocol described above for the measurement of SCR and Ca^2+^ leak was repeated using normal Tyrode or 0Na^+^/0Ca^2+^ Tyrode solution supplemented with AR antagonist (1 μM) and NE (1 μM).

### Statistical analysis

All values were represented as mean±SEM. All statistical analyses were performed by Student’s *t* test (two-tailed) or one-way ANOVA followed by the Tukey-Kramer post-hoc study for multiple comparisons. P value <0.05 was considered to indicate statistical significance in this study.

## Results

### Norepinephrine strikingly elongates the duration of AF

**Fig 1:**
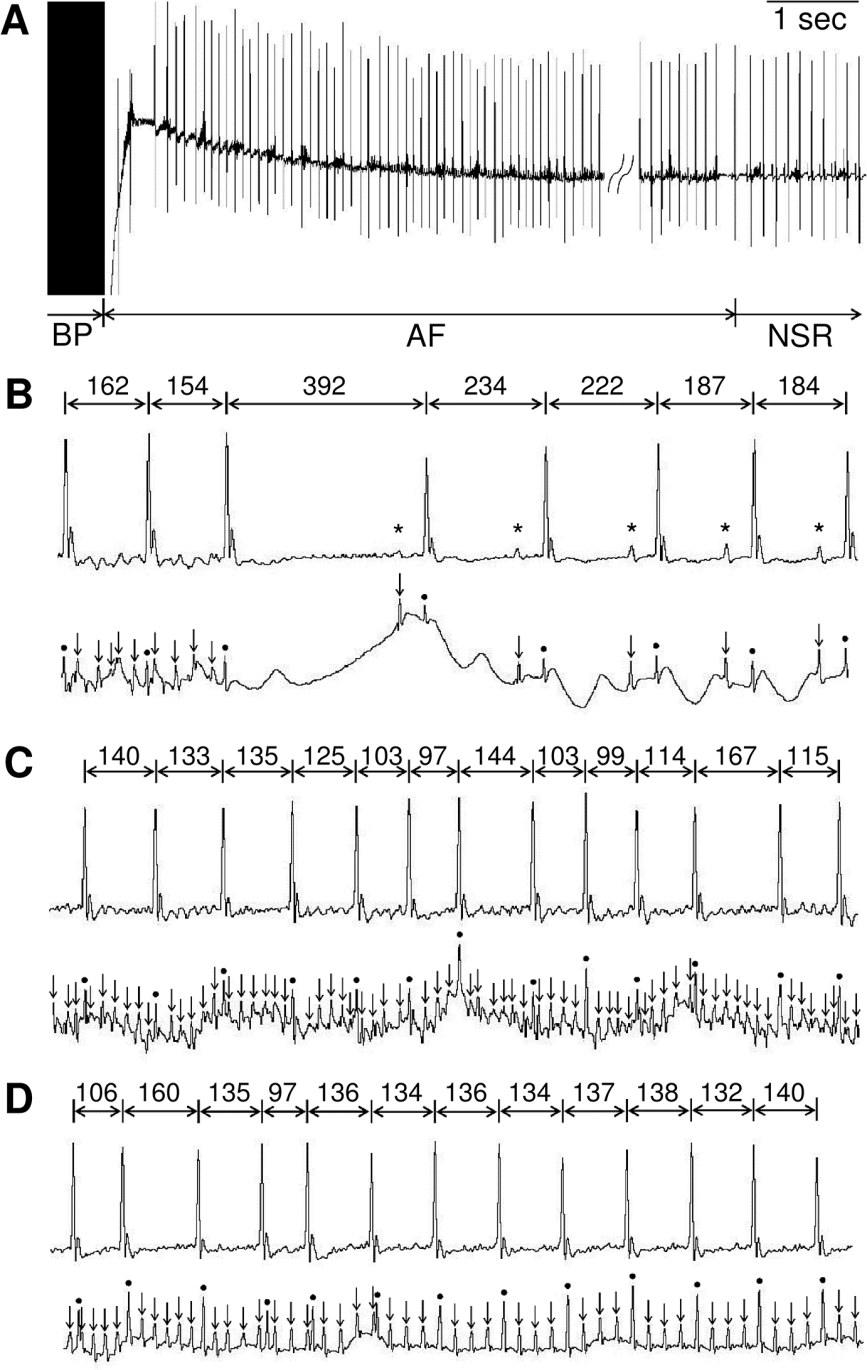
Induction of AF by transesophageal atrial burst pacing in mice. Representative lead II body surface electrocardiogram (ECG) recordings **(A).** Simultaneous recordings of Lead II body surface ECG (upper) and esophageal ECG (lower) **(B-D)**. **(A)** AF was induced by transesophageal atrial burst pacing (BP). An AF lasted about 32 seconds before spontaneous conversion into normal sinus rhythm (NSR). **(B)** Spontaneous conversion from AF to NSR. **(C)** Representative example of AF episode with disorganized fibrillatory atrial activities and irregular ventricular responses. **(D)** Conversion from AF to Afl with 4:1 atrioventricular-nodal conduction. Asterisks, arrows and circles indicate P-waves, atrial- and ventricular-electrograms, respectively. All R-R intervals are expressed in milliseconds.

**Fig 2:**
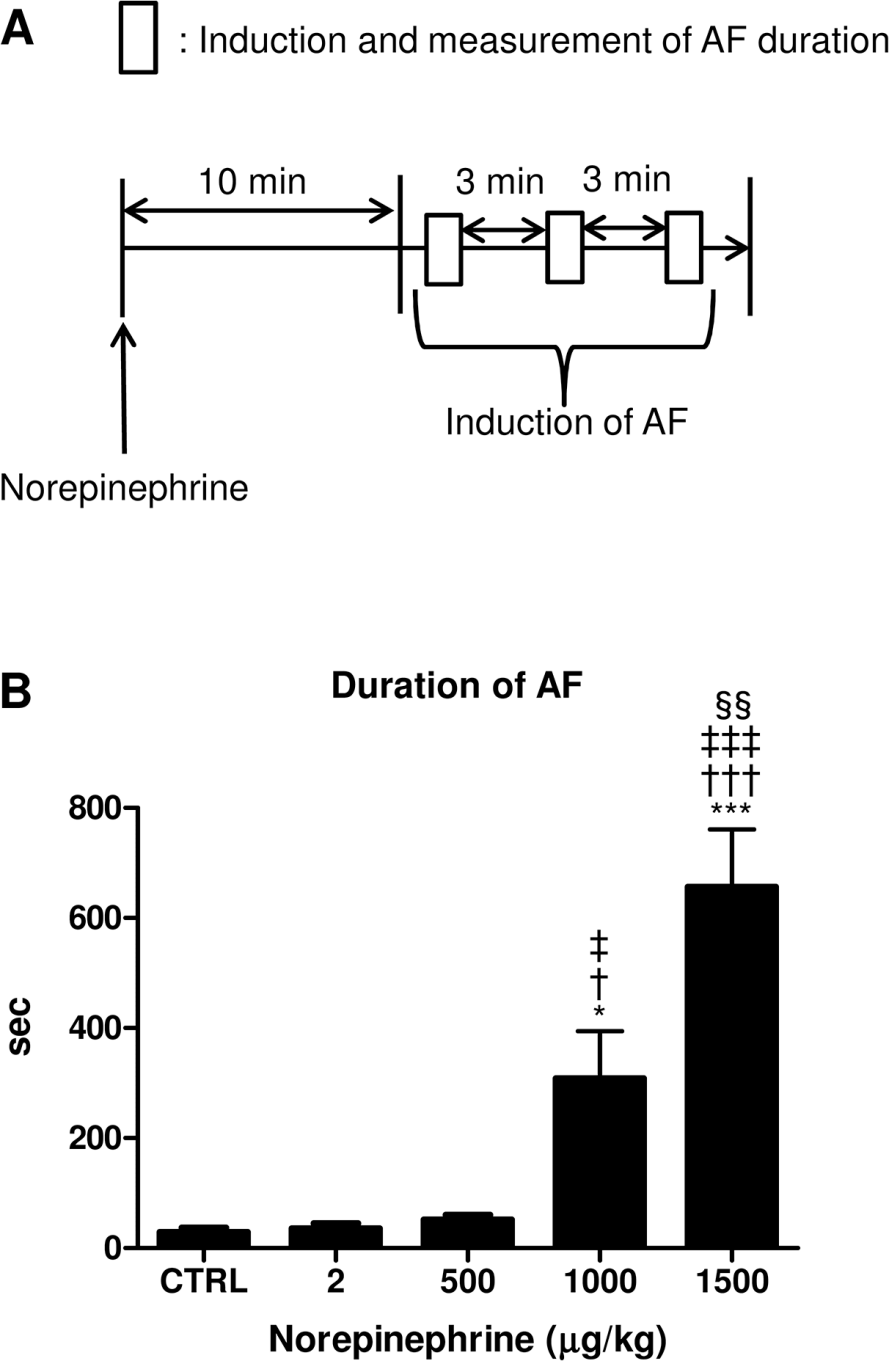
Norepinephrine strikingly elongates the duration of AF. **(A)** Schematic diagrams of experimental protocol to induce AF after sympathetic activation in mice. Mice were treated with 1.5 mg/kg of norepinephrine (NE) by intraperitoneal injection followed by transesophageal atrial burst pacing to induce AF. The rectangle represents the period from the start of burst pacing to the termination of AF. Note that, for each individual animal, the longest duration among 10 trials was taken to be the duration of AF after NE administration. **(B)** The duration of AF was strikingly increased after NE administration in a dose-dependent manner. NE was intraperitoneally injected into each mouse at one of several doses as indicated below, and was followed by transesophageal atrial burst pacing (n = 6–8, *P<0.05 vs CTRL, †P<0.05 vs 2 μg/kg, ‡P<0.05 vs 500 μg/kg) (n = 6–8, ***P<0.001 vs CTRL, †††P<0.001 vs 2 μg/kg, ‡‡‡P<0.001 vs 500 μg/kg, §§P<0.01 vs 1000 μg/kg).

AF was inducible by trans-esophageal pacing, as previously reported [10]. AF was defined as an irregular heart rhythm with loss of P-waves lasting at least 2 sec before spontaneous conversion into normal sinus rhythm (Fig 1A and 1B) [15,16]. During pacing-induced arrhythmic events, however, there occurred not only AF, but also intermittent regular atrial activities (Fig 1C and 1D), which is most likely atrial flutter (Afl). We thus measured the time from the end of burst pacing to spontaneous conversion into normal sinus rhythm (NSR), which indeed included both AF and Afl. Further, such duration was not always identical among different pacings. We thus performed three series of burst pacing over a 3-minute interval for each mouse, and the duration of the longest AF was used as index in this study (Fig 2A).

With the above-mentioned method, AF was reliably induced, but with a short duration, as pointed out in previous studies [7,17]. The duration was only a few to tens of seconds (Figs 1 and 2B). With such a short duration, we thought that it will be difficult to convincingly examine the effect of pharmacological stimulation.

Because it has been suggested, in human, that autonomic imbalance may trigger the onset and duration of AF, we investigated the effect of adrenergic activation by intra-peritoneal NE injection. We found that NE administration strikingly and stably elongated the duration of AF in a dose-dependent manner (Fig 2B). The AF duration was less than 30 seconds (29.0±8.1 sec) in the absence of NE, but increased to 35.7±9.4 sec with NE (2 μg/kg), 51.8±8.3 sec (500 μg/kg), 308.3±86.2 sec (1 mg/kg), and 656.2±104.8 sec (1.5 mg/kg). Thus, the duration was increased by more than 20-fold, to more than 10 minutes.

### Norepinephrine elongates AF duration through β1- and α1- adrenergic receptor-mediated signaling

**Fig 3:**
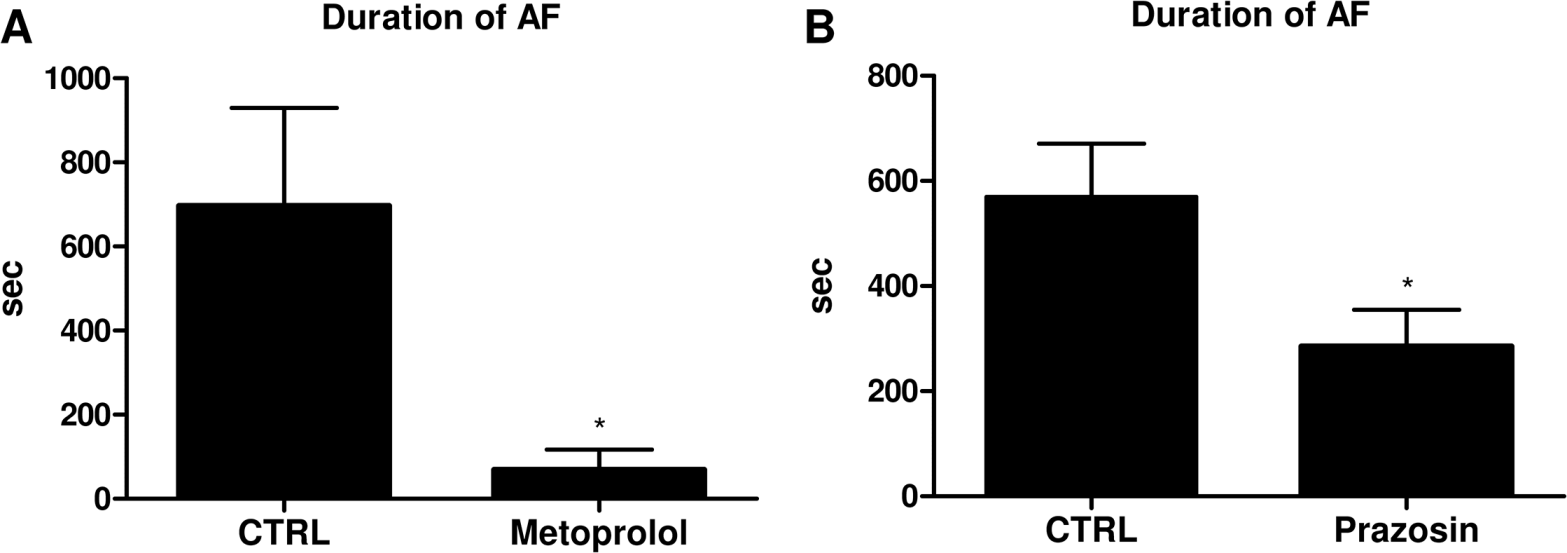
Norepinephrine elongates AF duration through β1- and α1-adrenergic receptor-mediated signaling. **(A)** 2 mg/kg of metoprolol, **(B)** 1 mg/kg of prazosin or natural saline (CTRL) was intraperitoneally injected into conscious mice 45 min before the administration of NE (1.5 mg/kg). Both metoprolol and prazosin treatment significantly shortened the NE-elongated AF. (n = 10–13, *P<0.05, #P<0.1 vs CTRL) (n = 10–15, *P<0.05).

To determine which types of AR-mediated signaling play important roles in the NE-induced elongation of AF, we examined the effects of prazosin and metoprolol on the duration of AF in our model. The doses of metoprolol and prazosin were determined based on previous reports [18,19]. The heart rate (HR) just before the AF induction by burst pacing was significantly lower in metoprolol treated group compared with the control group (control 490.6±11.0 sec vs metoprolol 421.4±30.4 sec, P<0.05). On the other hand, no significant difference was observed in the HR between prazosin treated group and control group (control 504.4±11.3 sec vs prazosin 481.9±8.7 sec, not significant). The duration of the AF was significantly shortened by both metoprolol (control 696.6±232.8 sec vs metoprolol 69.1±47.6 sec, P<0.05) (Fig 3A) and prazosin (control 569.7±101.1 sec vs prazosin 285.2±69.6 sec, P<0.05) (Fig 3B), indicating that both β1-AR and α1-AR signaling pathways play important roles in the NE-induced elongation of AF.

### Norepinephrine induces SR Ca^2+^ leak and spontaneous Ca^2+^ releases via β1- and α1-AR-mediated signaling in atrial myocytes

**Fig 4:**
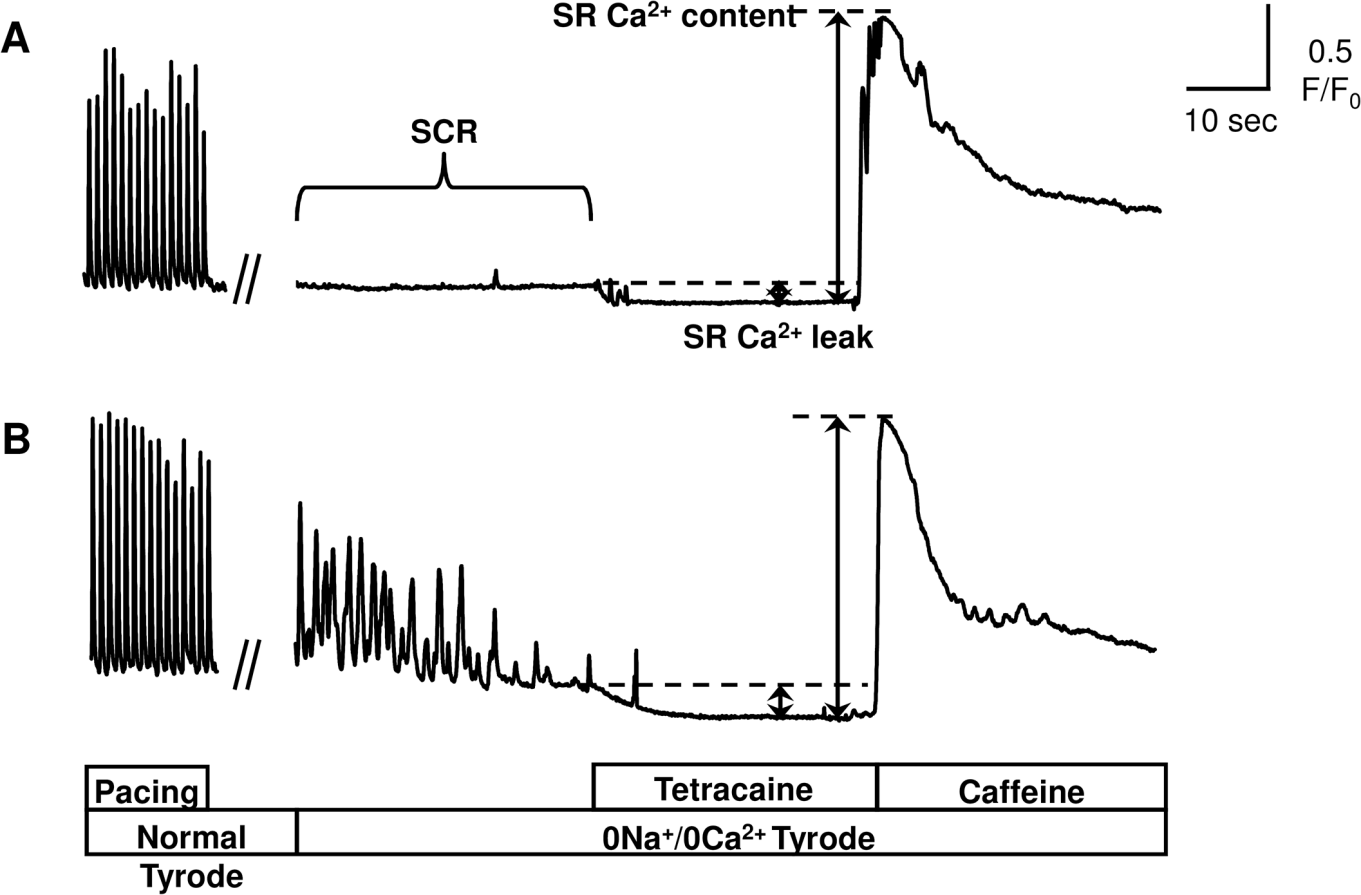
Measurement of Ca^2+^ transient in atrial myocytes. Representative Ca^**2+**^ traces of atrial myocytes in the absence **(A)** or presence **(B)** of 1 μM NE. Fluo-4 loaded myocytes were electrically paced at 1 Hz for 15 seconds followed by a rapid switch of the extracellular solution from normal Tyrode to 0Na^**+**^/0Ca^**2+**^ Tyrode. The spontaneous Ca^**2+**^ release (SCR) was counted for 30 seconds. The diastolic Ca^**2+**^ leak from sarcoplasmic reticulum (SR), SR Ca^**2+**^ leak, was estimated by measuring the downward shift in fluorescence after 1 mM tetracaine treatment. Finally, 10 mM caffeine was administered rapidly to estimate the SR Ca^**2+**^ content.

**Fig 5:**
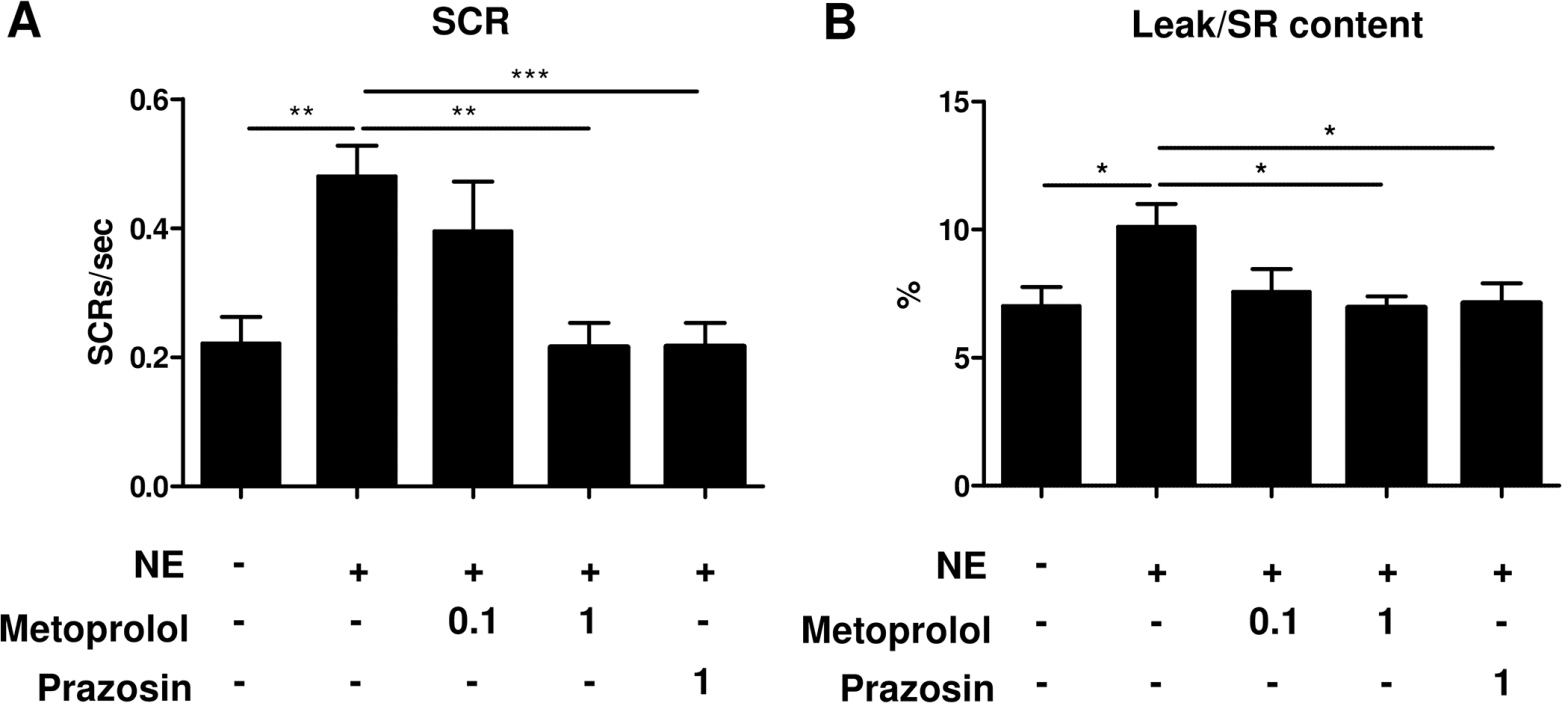
Norepinephrine induces SR Ca^2+^ leak and spontaneous Ca^2+^ releases via β1- and α1-AR mediated signaling. Quantification of spontaneous Ca^**2+**^ release **(A)** and SR Ca^**2+**^ leak **(B)**. Atrial myocytes were incubated with metoprolol or prazosin in the absence (-) or presence (+) of 1 μM NE. Metoprolol and prazosin significantly reversed the NE-enhanced SCR (n = 14–22, **P<0.01, ***P<0.001) and SR Ca^**2+**^ leak (n = 14–22, *P<0.05). The magnitude of SR Ca^**2+**^ leak is expressed as a relative value to SR Ca^**2+**^ content. Values under the graph represent the concentrations of adrenergic receptor antagonists (μM).

To elucidate the mechanism underlying the adrenergic activation-induced elongation of AF, we next examined the effect of NE on the SR Ca^2+^ leak and SCR in isolated atrial myocytes. The magnitude of the diastolic Ca^2+^ leak from SR was expressed as a value relative to the caffeine-releasable SR Ca^2+^ content [20]. The adrenergic activation by NE increased the SR Ca^2+^ leak (~1.4-fold compared to control, P<0.05) and the rate of SCR (~2.1-fold, P<0.01) (Fig 4A and 4B). Consistent with the findings of our in-vivo study (Fig 3), treatment with 1 μM metoprolol significantly suppressed the NE-induced increase in the SR Ca^2+^ leak (~31% lower than that seen in untreated myocytes, P<0.05) and SCR (~54% lower, P<0.01) in a dose-dependent manner. In addition, prazosin treatment also significantly attenuated the SR Ca^2+^ leak (~30% lower, P<0.05) and SCR (~55% lower, P<0.001) (Fig 5A and 5B). These results suggest that not only β1-AR but also α1-AR-mediated signaling are involved in the NE-induced SR Ca^2+^ leak and SCR in atrial myocytes.

## Discussion

In this study, we established an adrenergic activation-induced long-lasting AF model in mice. NE injection significantly elongated transesophageal burst pacing-induced AF from around 30 seconds [7] to 10 minutes. By this method we were able to stably induce, what is to our knowledge, the longest AF ever reported in genetically-unmodified mice. The development of AF has been demonstrated by genetic overexpression of several molecules that are involved in β-AR or α-AR mediated signaling [21], such as cAMP-response element modulator [22], Gαq [23], or Rho A [24]. However, it has been desired to induce AF in genetically-unmodified normal mice. In this regard, the short duration of AF in genetically-unmodified animal models has been a major problem in investigating the mechanisms of AF. Long-term observation of AF episodes in our model will enable us to examine in greater detail the mechanisms involved in AF maintenance, such as AF-induced atrial remodeling [25]. In addition, it will provide researchers with time to inject potentially useful drugs after the onset of AF, in order to evaluate the efficiency of such a drug for defibrillation of AF. Furthermore, this is a minimally invasive model, requiring no surgical procedure *e*.*g*. intravascular catheterization [26]. Thus, we can repetitively examine susceptibility to AF over a long observation period. Additionally, the applicability of this model to genetically-modified mice will enable us to obtain more solid evidence of the importance of specific molecules in AF development.

Using the model, we next investigated which AR subtype was more prominently involved in AF maintenance. The sympathetic and parasympathetic nervous systems play pivotal roles in the development of AF [8]. Consistently, activation of the sympathetic and parasympathetic systems is observed before the onset of AF [27]. In addition, autonomic nervous system function is thought to be involved in the arrhythmogenic mechanisms of several risk factors underlying AF including hyperthyroidism, exercise and ischemic heart disease [28–30]. Moreover, atrial tachyarrhythmias can be induced by activating the mediastinal nerves, which causes activation of the sympathetic and parasympathetic systems in the heart [31]. Based on these findings, the inhibition of inappropriate autonomic nervous system activation has been adopted to prevent development of AF. As expected, the usefulness of β1-AR blockers [32,33] and autonomic denervation [34] have been demonstrated in several human studies. Yet the efficacy of those treatments is limited, at least in part because of our incomplete understanding of the mechanisms underlying autonomic activation-induced atrial arrhythmogenesis. Thus the identification of the signaling pathway that is predominantly involved in catecholamine-induced arrhythmogenesis is an important step toward developing more effective strategies for AF prevention. Consistent with previous reports [35], a β1-AR-specific blocker was effective at preventing NE-induced elongation of AF in our mouse model. In addition, an α1-AR-specific blocker also shortened the NE-induced elongation of AF. These findings support the recently proposed concept that both α-AR and β-AR play important roles in the development of AF.

To further investigate the mechanism by which both types of receptor-mediated signaling contribute to maintaining AF, we assessed the SR Ca^2+^ leak and the consequent spontaneous SR Ca^2+^ release in cultured atrial cardiomyocytes. Catecholamine-induced phosphorylation of RYR by protein kinase A or Ca^2+^/calmodulin-dependent protein kinase II (CaMKII) is reported to cause diastolic SR Ca^2+^ leak, leading to delayed afterdepolarization (DAD), which is recognized as a major source of ectopic activity [8,36]. Ectopic activity is accepted as one of the major mechanisms responsible for the onset and maintenance of AF [8]. β-AR activation has been reported to induce SR Ca^2+^ leak and spontaneous SR Ca^2+^ release [37]. Consistently, the β1-AR-specific blocker metoprolol attenuated the NE-induced SR Ca^2+^ leak and the spontaneous SR Ca^2+^ release. In keeping with the results of our in-vivo study, the α1-AR-specific blocker prazosin had a similar effect.

Recent reports have documented an important role of α1-AR-mediated signaling including Gq, phospholipase C, inositol triphosphate receptor (IP3R), protein kinase C, and CaMKII in the regulation of Ca^2+^ transient in cardiomyocytes [38–40]. In addition, endothelin, which also elicits IP3R, Ca^2+^, and CaMKII-mediated signaling, has been reported to induce elevation of intracellular Ca^2+^ concentration through SR Ca^2+^ release from IP3R, leading to spontaneous Ca^2+^ release from RYR in atrial myocytes [41]. Thus the α1-AR activation-induced SR Ca^2+^ leak that was observed in the present study may have been caused by a similar mechanism.

On the other hand, it has been reported that α-AR signaling induces the activation of cardiac neurons [42,43]. In addition, α-adrenergic activation can inhibit inwardly rectifying K^+^ current, thereby enhancing automaticity [44]. These factors can be also considered among the candidate mechanisms that may be responsible for α1 AR activation-induced AF.

These findings imply that consideration of α1-AR-mediated signaling may also be important in the management of AF. Along the same lines, a recent report showed that the α, β-blocker carvedilol is more useful than the β1 selective blocker metoprolol in preventing AF after cardiac surgery [45].

A possible limitation of this study is that we measured Ca^2+^ transient at room temperature following the method of previous reports [13,14]. The temperature might have affected the response of cardiomyocytes.

In conclusion, we established an adrenergic activation-induced long-lasting AF model in mice. Using the model, we demonstrated the important role of β1- and α1-AR-mediated signaling in the maintenance of AF. In addition, we showed that not only β1-AR but also α1-AR activation are involved in the SR Ca^2+^ leak in atrial cardiomyocytes. This model and the knowledge we have obtained through its use will be useful in establishing novel therapeutic targets and agents for the treatment of AF.

## References

[1] Ortiz J, Niwano S, Abe H, Rudy Y, Johnson NJ, Waldo AL (1994) Mapping the conversion of atrial flutter to atrial fibrillation and atrial fibrillation to atrial flutter. Insights into mechanisms. Circ Res 74: 882–894. 8156635 10.1161/01.res.74.5.882

[2] January CT, Wann LS, Alpert JS, Calkins H, Cigarroa JE, Cleveland JC Jr, et al (2014) 2014 AHA/ACC/HRS Guideline for the Management of Patients With Atrial Fibrillation: A Report of the American College of Cardiology/American Heart Association Task Force on Practice Guidelines and the Heart Rhythm Society. J Am Coll Cardiol 64: e1–e76. 10.1016/j.jacc.2014.03.022 24685669

[3] Heeringa J, van der Kuip DA, Hofman A, Kors JA, van Herpen G, Stricker BH, et al (2006) Prevalence, incidence and lifetime risk of atrial fibrillation: the Rotterdam study. Eur Heart J 27: 949–953. 16527828 10.1093/eurheartj/ehi825

[4] Lloyd-Jones DM, Wang TJ, Leip EP, Larson MG, Levy D, Vasan RS, et al (2004) Lifetime risk for development of atrial fibrillation: the Framingham Heart Study. Circulation 110: 1042–1046. 15313941 10.1161/01.CIR.0000140263.20897.42

[5] Piccini JP, Hammill BG, Sinner MF, Jensen PN, Hernandez AF, Heckbert SR, et al (2012) Incidence and prevalence of atrial fibrillation and associated mortality among Medicare beneficiaries, 1993–2007. Circ Cardiovasc Qual Outcomes 5: 85–93. 10.1161/CIRCOUTCOMES.111.962688 22235070 PMC3332107

[6] January CT, Wann LS, Alpert JS, Calkins H, Cigarroa JE, Cleveland JC Jr, et al (2014) 2014 AHA/ACC/HRS Guideline for the Management of Patients With Atrial Fibrillation: Executive Summary: A Report of the American College of Cardiology/American Heart Association Task Force on Practice Guidelines and the Heart Rhythm Society. Circulation 130: 2071–2104. 10.1161/CIR.0000000000000040 24682348

[7] Schrickel JW, Bielik H, Yang A, Schimpf R, Shlevkov N, Burkhardt D, et al (2002) Induction of atrial fibrillation in mice by rapid transesophageal atrial pacing. Basic Res Cardiol 97: 452–460. 12395207 10.1007/s003950200052

[8] Chen PS, Chen LS, Fishbein MC, Lin SF, Nattel S (2014) Role of the autonomic nervous system in atrial fibrillation: pathophysiology and therapy. Circ Res 114: 1500–1515. 10.1161/CIRCRESAHA.114.303772 24763467 PMC4043633

[9] Verheule S, Sato T, Everett Tt, Engle SK, Otten D, Rubart-von der Lohe M, et al (2004) Increased vulnerability to atrial fibrillation in transgenic mice with selective atrial fibrosis caused by overexpression of TGF-beta1. Circ Res 94: 1458–1465. 15117823 10.1161/01.RES.0000129579.59664.9d PMC2129102

[10] Haugan K, Lam HR, Knudsen CB, Petersen JS (2004) Atrial fibrillation in rats induced by rapid transesophageal atrial pacing during brief episodes of asphyxia: a new in vivo model. J Cardiovasc Pharmacol 44: 125–135. 15175567 10.1097/00005344-200407000-00017

[11] Chelu MG, Sarma S, Sood S, Wang S, van Oort RJ, Skapura DG, et al (2009) Calmodulin kinase II-mediated sarcoplasmic reticulum Ca2+ leak promotes atrial fibrillation in mice. J Clin Invest 119: 1940–1951. 19603549 10.1172/JCI37059 PMC2701862

[12] Rose RA, Kabir MG, Backx PH (2007) Altered heart rate and sinoatrial node function in mice lacking the cAMP regulator phosphoinositide 3-kinase-gamma. Circ Res 101: 1274–1282. 17975110 10.1161/CIRCRESAHA.107.158428

[13] Watanabe H, Chopra N, Laver D, Hwang HS, Davies SS, Roach DE, et al (2009) Flecainide prevents catecholaminergic polymorphic ventricular tachycardia in mice and humans. Nat Med 15: 380–383. 10.1038/nm.1942 19330009 PMC2904954

[14] Shannon TR, Pogwizd SM, Bers DM (2003) Elevated sarcoplasmic reticulum Ca2+ leak in intact ventricular myocytes from rabbits in heart failure. Circ Res 93: 592–594. 12946948 10.1161/01.RES.0000093399.11734.B3

[15] Pretorius L, Du XJ, Woodcock EA, Kiriazis H, Lin RC, Marasco S, et al (2009) Reduced phosphoinositide 3-kinase (p110alpha) activation increases the susceptibility to atrial fibrillation. Am J Pathol 175: 998–1009. 10.2353/ajpath.2009.090126 19679877 PMC2731119

[16] Sapra G, Tham YK, Cemerlang N, Matsumoto A, Kiriazis H, Bernardo BC, et al (2014) The small-molecule BGP-15 protects against heart failure and atrial fibrillation in mice. Nat Commun 5: 5705 10.1038/ncomms6705 25489988

[17] Fukui A, Takahashi N, Nakada C, Masaki T, Kume O, Shinohara T, et al (2013) Role of leptin signaling in the pathogenesis of angiotensin II-mediated atrial fibrosis and fibrillation. Circ Arrhythm Electrophysiol 6: 402–409. 10.1161/CIRCEP.111.000104 23406575

[18] Sato S (2008) Quantitative evaluation of ontogenetic change in heart rate and its autonomic regulation in newborn mice with the use of a noninvasive piezoelectric sensor. Am J Physiol Heart Circ Physiol 294: H1708–1715. 10.1152/ajpheart.01122.2007 18263713

[19] Gross V, Tank J, Obst M, Plehm R, Blumer KJ, Diedrich A, et al (2005) Autonomic nervous system and blood pressure regulation in RGS2-deficient mice. Am J Physiol Regul Integr Comp Physiol 288: R1134–1142. 15661972 10.1152/ajpregu.00246.2004

[20] Shan J, Betzenhauser MJ, Kushnir A, Reiken S, Meli AC, Wronska A, et al (2010) Role of chronic ryanodine receptor phosphorylation in heart failure and beta-adrenergic receptor blockade in mice. J Clin Invest 120: 4375–4387. 10.1172/JCI37649 21099115 PMC2993577

[21] Riley G, Syeda F, Kirchhof P, Fabritz L (2012) An introduction to murine models of atrial fibrillation. Front Physiol 3: 296 10.3389/fphys.2012.00296 22934047 PMC3429067

[22] Muller FU, Lewin G, Baba HA, Boknik P, Fabritz L, Kirchhefer U, et al (2005) Heart-directed expression of a human cardiac isoform of cAMP-response element modulator in transgenic mice. J Biol Chem 280: 6906–6914. 15569686 10.1074/jbc.M407864200

[23] Hirose M, Takeishi Y, Niizeki T, Shimojo H, Nakada T, Kubota I, et al (2009) Diacylglycerol kinase zeta inhibits G(alpha)q-induced atrial remodeling in transgenic mice. Heart Rhythm 6: 78–84. 10.1016/j.hrthm.2008.10.018 19121805

[24] Sah VP, Minamisawa S, Tam SP, Wu TH, Dorn GW 2nd, Ross J Jr, et al (1999) Cardiac-specific overexpression of RhoA results in sinus and atrioventricular nodal dysfunction and contractile failure. J Clin Invest 103: 1627–1634. 10377168 10.1172/JCI6842 PMC408391

[25] Heijman J, Voigt N, Nattel S, Dobrev D (2014) Cellular and molecular electrophysiology of atrial fibrillation initiation, maintenance, and progression. Circ Res 114: 1483–1499. 10.1161/CIRCRESAHA.114.302226 24763466

[26] Wakimoto H, Maguire CT, Kovoor P, Hammer PE, Gehrmann J, Triedman JK, et al (2001) Induction of atrial tachycardia and fibrillation in the mouse heart. Cardiovasc Res 50: 463–473. 11376622 10.1016/s0008-6363(01)00264-4

[27] Tan AY, Zhou S, Ogawa M, Song J, Chu M, Li H, et al (2008) Neural mechanisms of paroxysmal atrial fibrillation and paroxysmal atrial tachycardia in ambulatory canines. Circulation 118: 916–925. 10.1161/CIRCULATIONAHA.108.776203 18697820 PMC2742977

[28] Stavrakis S, Yu X, Patterson E, Huang S, Hamlett SR, Chalmers L, et al (2009) Activating autoantibodies to the beta-1 adrenergic and m2 muscarinic receptors facilitate atrial fibrillation in patients with Graves' hyperthyroidism. J Am Coll Cardiol 54: 1309–1316. 10.1016/j.jacc.2009.07.015 19778674 PMC2801559

[29] Andrade J, Khairy P, Dobrev D, Nattel S (2014) The clinical profile and pathophysiology of atrial fibrillation: relationships among clinical features, epidemiology, and mechanisms. Circ Res 114: 1453–1468. 10.1161/CIRCRESAHA.114.303211 24763464

[30] Rathore SS, Berger AK, Weinfurt KP, Schulman KA, Oetgen WJ, Gersh BJ, et al (2000) Acute myocardial infarction complicated by atrial fibrillation in the elderly: prevalence and outcomes. Circulation 101: 969–974. 10704162 10.1161/01.cir.101.9.969

[31] Armour JA, Richer LP, Page P, Vinet A, Kus T, Vermeulen M, et al (2005) Origin and pharmacological response of atrial tachyarrhythmias induced by activation of mediastinal nerves in canines. Auton Neurosci 118: 68–78. 15795179 10.1016/j.autneu.2005.01.006

[32] Van Noord T, Tieleman RG, Bosker HA, Kingma T, Van Veldhuisen DJ, Crijns HJ, et al (2004) Beta-blockers prevent subacute recurrences of persistent atrial fibrillation only in patients with hypertension. Europace 6: 343–350. 15172659 10.1016/j.eupc.2004.04.001

[33] Yoshioka I, Sakurai M, Namai A, Kawamura T (2009) Postoperative treatment of carvedilol following low dose landiolol has preventive effect for atrial fibrillation after coronary artery bypass grafting. Thorac Cardiovasc Surg 57: 464–467. 10.1055/s-0029-1186069 20013619

[34] Katritsis DG, Pokushalov E, Romanov A, Giazitzoglou E, Siontis GC, Po SS, et al (2013) Autonomic denervation added to pulmonary vein isolation for paroxysmal atrial fibrillation: a randomized clinical trial. J Am Coll Cardiol 62: 2318–2325. 10.1016/j.jacc.2013.06.053 23973694

[35] Marks AR (2013) Calcium cycling proteins and heart failure: mechanisms and therapeutics. J Clin Invest 123: 46–52. 10.1172/JCI62834 23281409 PMC3533269

[36] Wakili R, Voigt N, Kaab S, Dobrev D, Nattel S (2011) Recent advances in the molecular pathophysiology of atrial fibrillation. J Clin Invest 121: 2955–2968. 10.1172/JCI46315 21804195 PMC3148739

[37] Ogrodnik J, Niggli E (2010) Increased Ca(2+) leak and spatiotemporal coherence of Ca(2+) release in cardiomyocytes during beta-adrenergic stimulation. J Physiol 588: 225–242. 10.1113/jphysiol.2009.181800 19900959 PMC2821561

[38] Zeng Z, Zhang H, Lin N, Kang M, Zheng Y, Li C, et al (2014) Role of Inositol-1,4,5-Trisphosphate Receptor in the Regulation of Calcium Transients in Neonatal Rat Ventricular Myocytes. J Pharmacol Sci 126: 37–46. 25242084

[39] O-Uchi J, Sasaki H, Morimoto S, Kusakari Y, Shinji H, Obata T, et al (2008) Interaction of alpha1-adrenoceptor subtypes with different G proteins induces opposite effects on cardiac L-type Ca2+ channel. Circ Res 102: 1378–1388. 10.1161/CIRCRESAHA.107.167734 18467629

[40] Grimm M, El-Armouche A, Zhang R, Anderson ME, Eschenhagen T (2007) Reduced contractile response to alpha1-adrenergic stimulation in atria from mice with chronic cardiac calmodulin kinase II inhibition. J Mol Cell Cardiol 42: 643–652. 17292391 10.1016/j.yjmcc.2006.12.010

[41] Zima AV, Blatter LA (2004) Inositol-1,4,5-trisphosphate-dependent Ca(2+) signalling in cat atrial excitation-contraction coupling and arrhythmias. J Physiol 555: 607–615. 14754996 10.1113/jphysiol.2003.058529 PMC1664857

[42] Armour JA (1997) Intrinsic cardiac neurons involved in cardiac regulation possess alpha 1-, alpha 2-, beta 1- and beta 2-adrenoceptors. Can J Cardiol 13: 277–284. 9117916

[43] Ishibashi H, Umezu M, Jang IS, Ito Y, Akaike N (2003) Alpha 1-adrenoceptor-activated cation currents in neurones acutely isolated from rat cardiac parasympathetic ganglia. J Physiol 548: 111–120. 12598585 10.1113/jphysiol.2002.033100 PMC2342805

[44] Sato R, Koumi S (1995) Modulation of the inwardly rectifying K+ channel in isolated human atrial myocytes by alpha 1-adrenergic stimulation. J Membr Biol 148: 185–191. 8606367 10.1007/BF00207274

[45] Wang HS, Wang ZW, Yin ZT (2014) Carvedilol for prevention of atrial fibrillation after cardiac surgery: a meta-analysis. PLoS One 9: e94005 10.1371/journal.pone.0094005 24705913 PMC3976381

